# Supplementary material for: miR-126-3p and miR-21-5p as Hallmarks of Bio-Positive Ageing; Correlation Analysis and Machine Learning Prediction in Young to Ultra-Centenarian Sicilian Population
Source: Cells. 2022 Apr 30;11(9):1505. doi: 10.3390/cells11091505 (PMC9099697; doi:10.3390/cells11091505)
Supplement: Supplementary file 1 [file cells-11-01505-s001.zip › cells-1626674-supplementary.pdf]

Supplemental Table S1. Anthropometric, hematological, hematochemical, molecular, oxidative stress parameters and smoking habits.

**Variable (unit of measurement)**	**(a) Young adults (22-50 y.o.) N=19, M=7, W=12**	**(b) Adults (51-70 y.o.) N=28, M=14, W=14**	**(c) Older adults (71-99 y.o.) N=20, M=13, W=7**	**(d) Ultra-centenarians (100-111 y.o.) N=11, M=2, W=9**	***P***
BMI	21.60 (17.30-30.20)<br>N=19, (22.49±3.57)<br>M=7, (25.21±2.52)<br>W=12, (20.90±3.14)	27.20 (21.40-35.40)<br>N=27, (27.29±3.32)<br>M=14, (27.41±2.61)<br>W=13, (27.15±4.05)	29.00 (18.70-40.90)<br>N=20, (28.84±5.59)<br>M=13, (28.82±4.72)<br>W=7, (28.86 ±7.37)	22.30 (18.40-29.70)<br>N=10, (22.95±3.09)<br>M=2, (26.10±5.09)<br>W=8, (22.16 ±2.25)	a vs b,c<br>d vs b,c
Hematochemical parameter					
CRP (mg/dL, <5)	0.63 (0.16-23.23)<br>N=19, (2.67±5.54)<br>M=7, (0.60±0.55)<br>W=12, (3.88±6.76)	1.68 (0.42-63.86)<br>N=27, (4.32±11.97)<br>M=14, (5.90±16.71)<br>W=13, (2.61±1.41)	1.10 (0.23-26.75)<br>N=20 (3.19±5.88)<br>M=13, (3.40±7.07)<br>W=7, (2.79±3.03)	1.92 (1.13-5.97)<br>N=11, (2.62±1.57)<br>M=2, (3.36±0.45)<br>W=9, (2.45±1.70)	ns
**Molecular test**					
RTL	1.19 (0.95-1.51)<br>N=19, (1.20±1.74)<br>M=7, (1.12±0.20)<br>W=12, (1.25±0.15)	1.05 (0.68-1.31)<br>N=27 (0.99±0.16)<br>M=14, (0.98±0.17)<br>W=13, (1.00±0.14)	0.69 (0.45-1.19)<br>N=20, (0.74±0.19)<br>M=13, (0.73±0.16)<br>W=7, (0.76±0.25)	0.73 (0.47-0.85)<br>N=10, (0.70±0.12)<br>M=2, (0.67±0.12)<br>W=8, (0.71±0.13)	a vs b,c,d<br>b vs c,d
**Oxidative stress parameters**					
PON (U/L)	122.81 (40.63-392.43)<br>N=19, (149.63±91.62)<br>M=7, (136.39±75.73)<br>W=12, (157.36±102.13)	91.42 (43.40-300.09)<br>N=27, (112.41±62.84)<br>M=14, (115.88±74.57)<br>W=13, (108.67±50.04)	100.65 (19.39-222.53)<br>N=19, (99.28±57.82)<br>M=12, (106.49±62.23)<br>W=7, (86.93±51.48)	78.02 (43.40-254.85)<br>N=10, (105.26±72.61)<br>M=2, (137.58±96.63)<br>W=8, (97.19±71.21)	ns
TEAC (mM)	3601.24 (2589.58-4721.66)<br>N=19, (3577.83±463.34)	3794.43 (1805.68-5485.35)<br>N=27, (3784.18±686.92)	3574.19 (2819.23-5572.38)<br>N=19, (3764.02±718.84)	3650.68 (2967.68-4658.10)<br>N=10, (3725.91±502.69)	ns

	M=7,<br>(3523.58±653.98)<br>W=12,<br>(3609.47±339.18)	M=14,<br>(3837.36±839.79)<br>W=13,<br>(3726.92±501.50)	M=12,<br>(3596.05±572.30)<br>W=7,<br>(4051.99±892.50)	M=2,<br>(3812.89±1195.31)<br>W=8,<br>(3704.17±343.64)	
MDA (μmol/L)	2.61 (1.19-4.52)<br>N=19, (2.76±1.02)<br>M=7, (3.23±0.84)<br>W=12, (2.48±1.04)	2.62 (0.95-4.45)<br>N=27, (2.67±1.03)<br>M=14, (2.62±0.92)<br>W=13, (2.71±1.17)	2.84 (0.64-4.18)<br>N=19, (2.63±1.02)<br>M=12, (2.98±0.95)<br>W=7, (2.04±0.91)	2.55 (1.12-5.78)<br>N=10, (2.77±1.37)<br>M=2, (2.55±0.25)<br>W=8, (2.82±1.55)	ns
**Smoking habits**					
Smoker	N=5, 26.32%<br>M=2, 28.57%<br>W=3, 25.00%	N=7, 29.19%<br>M=2, 18.18%<br>W=5, 38.46%	N=3, 15.00%<br>M=3, 23.08%<br>W=0, 0%	N=0, 0%<br>M=0, 0%<br>W=0, 0%	
Ex-smoker	N=2, 10.53%<br>M=0, 0%<br>W=2, 16.67%	N=10, 41.67%<br>M=6, 54.55%<br>W=4, 30.77%	N=10, 50.0%<br>M=8, 61.54%<br>W=2, 28.57%	N=0, 0%<br>M=0, 0%<br>W=0, 0%	p=0.001*
Never smoked	N=12, 63.16%<br>M=5, 71.43<br>W=7, 58.33%	N=7, 29.17%<br>M=3, 27.27%<br>W=4, 30.77%	N=7, 35.00%<br>N=2, 15.38%<br>W=5, 71.43%	N=11, 100%<br>M=2, 100%<br>W=9, 100%	

Abbreviations: y.o.=years old; N=total number of cases; M=men; W=women; SD=standard deviation; BMI=body mass index; CRP=C-reactive protein; RTL=relative telomere length; PON=paraoxonase; TEAC=trolox equivalent antioxidant capacity; MDA=malondialdehyde.

Data underlined are the median (min-max) of the total number of cases. Data between round brackets are mean values ± SD.

a, b, c, and d indicate, respectively, young adults, adults, older adults, and LLIs.

The table shows the pairwise comparisons between the different groups, *i.e.*, a, b, c, d. p-value≤0.05 is considered significant; ns=not significant.

Bonferroni test is used for pairwise comparisons.

*To test if there is difference in the smoking habit for Age Class is used the Fisher's exact test.

Data are presented both as median, minimum, maximum values, and mean±standard deviation (SD). For continuous variables, the one-way Analysis of Variance (ANOVA) is used to test if age produces a significant effect on the parameters. The Fisher test is considered to evaluate the significance of the results. For each statistically significant effect, we conduct a post hoc multiple comparison test using Bonferroni method. For categorical variables, we consider the chi-squared test or the Fisher exact test to compare differences between groups of age. All analyses were performed using Stata version 16.1 and all hypothesis testing are considered statistically significant for p≤ 0.05.

Supplemental Table S2. KEGG pathway of miR-21-5p and miR-126-3p.

KEGG pathway	Predicted miR-21-5p target genes	Predicted miR-126-3p target genes

hsa04022_cGMP-PKG_signaling_pathway	OPRD1 ATP2B4 KCNMA1 CALML4 EDNRA ATF2 ADCY1 MYLK3 ROCK2	CREB5 PDE5A MEF2D MEF2C
hsa04070_Phosphatidylinositol_signaling_system	CALML4 PIK3R3 INPP5K INPP5B DGKB	DGKD IPPK DGKH PPIP5K2
hsa04211_Longevity_regulating_pathway	SOD2 PIK3R3 ATF2 ADCY1 PPARGC1A	CREB5 FOXO3
hsa04213_Longevity_regulating_pathway	PIK3R3 ADCY1 HDAC2EIF4EBP	FOXO3
hsa04330_Notch_signaling_pathwa	APH1B DVL1 HDAC2	DTX4 DTX3L

Top 5 enriched KEGG pathway clustered by validated targets of miR-21-5p and miR-126-3p and corresponding target genes.

Supplemental Table S3. REACTOME pathway of miR-21-5p and miR-126-3p.

**REACTOME pathway**	**Predicted miR-21-5p target genes**	**Predicted miR-126-3p target genes**
R-HSA-9614085_FOXO-mediated transcription	CITED2PPARGC1A RBL2 SMAD4SOD2 YWHAZ	BCL6 FOXO3
R-HSA-400253_Circadian Clock	ARNTL ATF2 CLOCKHDAC2 PPARGC1A	CREM CRTC1 MEF2C MEF2D
R-HSA-9615017_FOXO-mediated transcription of oxidative stress	PPARGC1A SMAD4SOD2	FOXO3 PLXNA4
R-HSA-9013148_CDC42 GTPase cycle	ARHGAP31 ARHGEF9 GIT2 PAK3 SH3PXD2A	ARAP2 ARHGAP35 ARHGAP42 CDC42BPA FARP1 FNBP1L IQGAP1 MCF2L
R-HSA-449147_Signaling by Interleukins	BRWD1HDAC2ITGB2 OPRD1 PIK3R3 PPIA PSMD8 PSMF1 RAP1B SOD2 SRGAP1 STAT5A	

		TOLLIPUBE2V1 WIPF2 YWHAZ

Top 5 enriched REACTOME pathway clustered by validated targets of miR-21-5p and miR-126-3p and corresponding target genes.

Supplemental Table S4. KEGG pathway of miR-146a-5p and miR-181a-5p.

KEGG pathway	Predicted miR-146a-5p target genes	Predicted miR-181a-5p target genes
hsa05205_Proteoglycans_in_cancer	CBL ERBB4 FZD1 FZD8 HSPG2 IHH IQGAP1 MAPK13 MSN PIK3CA PLCE1 PPP1R12B PRKACA PRKCG SMAD2 WNT16	ANK3 CAMK2D CCND1 CDKN1A ESR1 FZD5 HPSE2 ITPR2 PRKACB RAC1 TP53 WNT5A
hsa04020_Calcium_signaling_pathway	ERBB4 GRIN2A HTR2C ITPKB MYLK3 NOS1 PHKA2 PLCD3 PLCE1 PRKACA PRKCG	ADORA2B CACNA1B CALM1 CAMK2D ITPR2 ORAI2 PHKG2 PRKACB
hsa04070_Phosphatidylinositol_signaling_system	BPNT2 CDS1 INPP4B ITPKB MTMR14 MTMR8 PIK3CA PLCE1 PRKCG SLC8A1	CALM1 ITPR2 MTMR7 PPIP5K1 PTEN
hsa04110_Cell_cycle	HDAC2 ORC4 RBX1 SMAD2 SMC1A TFDP2	ABL1 CCND1 CDC27 CDC6 CDKN1A MCM3 SKP1

hsa04114_Oocyte_meiosis	MAPK13 PGR PPP2R5E PRKACA RBX1 SMC1A	SKP2 SMC1B TP53 CALM1 CAMK2D CDC27 CPEB3 ITPR2 PRKACB RPS6KA6 SKP1 SMC1B SPDYE11 SPDYE17

Top 5 enriched KEGG pathway clustered by validated targets of miR-146a-5p and miR-181a-5p and corresponding target genes.

Supplemental Table S5. REACTOME pathway of miR-146a-5p and miR-181a-5p.

REACTOME pathway	Predicted miR-146a-5p target genes	Predicted miR-181a-5p target genes
R-HSA-4090294_SUMOylation of intracellular receptors	PGR SUMO3 UBE2I	VDR ESR1 NR1I2 PPARA
R-HSA-112316_Neuronal System	ARHGEF7 CACNB2 EPB41 ERBB4 GLUL KCNQ5 KCNS1 KCNS2 LRRTM2 NCALD NLGN3 NPTN PPFIA1 PPFIBP2 PPM1E PRKAB2 PRKACA PRKAR2B PRKCG SLC1A2 SLC38A2 SLC6A11 SLC6A4 SLITRK4 TSPOAP1 GRIN2A KCNA2 KCNK7 DLG2 LRRC49	CACNA1B CALM1 CAMK2D CHRNA9 GLRA2 GNAI2 GRIP2 KCND3 KCNJ6 KCNK10, LIN7C LRFN2 NTRK3 PRKACB RAC1 RPS6KA6 SLC1A3 SLC1A7 SLC6A1 SNAP25 SYN2 SYT2 GRIN2A KCNA2 KCNK7 DLG2 LRRC49
R-HSA-112315_Transmission across Chemical Synapses	ARHGEF7 CACNB2 ERBB4 GLUL GRIN2A NCALD PPFIA1 PPM1E PRKAB2 PRKACA PRKAR2B DLG2 PRKCG SLC1A2 SLC38A2 SLC6A11 SLC6A4 TSPOAP1	CACNA1B CALM1 CAMK2D CHRNA9 GLRA2 GNAI2 GRIN2B GRIP2 KCNJ6 LIN7C NPTN PRKACB RAC1 RPS6KA6 SLC1A3 SLC1A7

		SLC6A1 SNAP25 SYN2 DLG2
R-HSA-3232118_SUMOylation of transcription factors	PIAS1 UBE2I SUMO3 TFAP2A	FOXL2 PIAS2 TP53BP1
R-HSA-3215018_Processing and activation of SUMO	SUMO3 UBE2I	SENP2

Top 5 enriched REACTOME pathway clustered by validated targets of miR-146a-5p and miR-181a-5p and corresponding target genes.

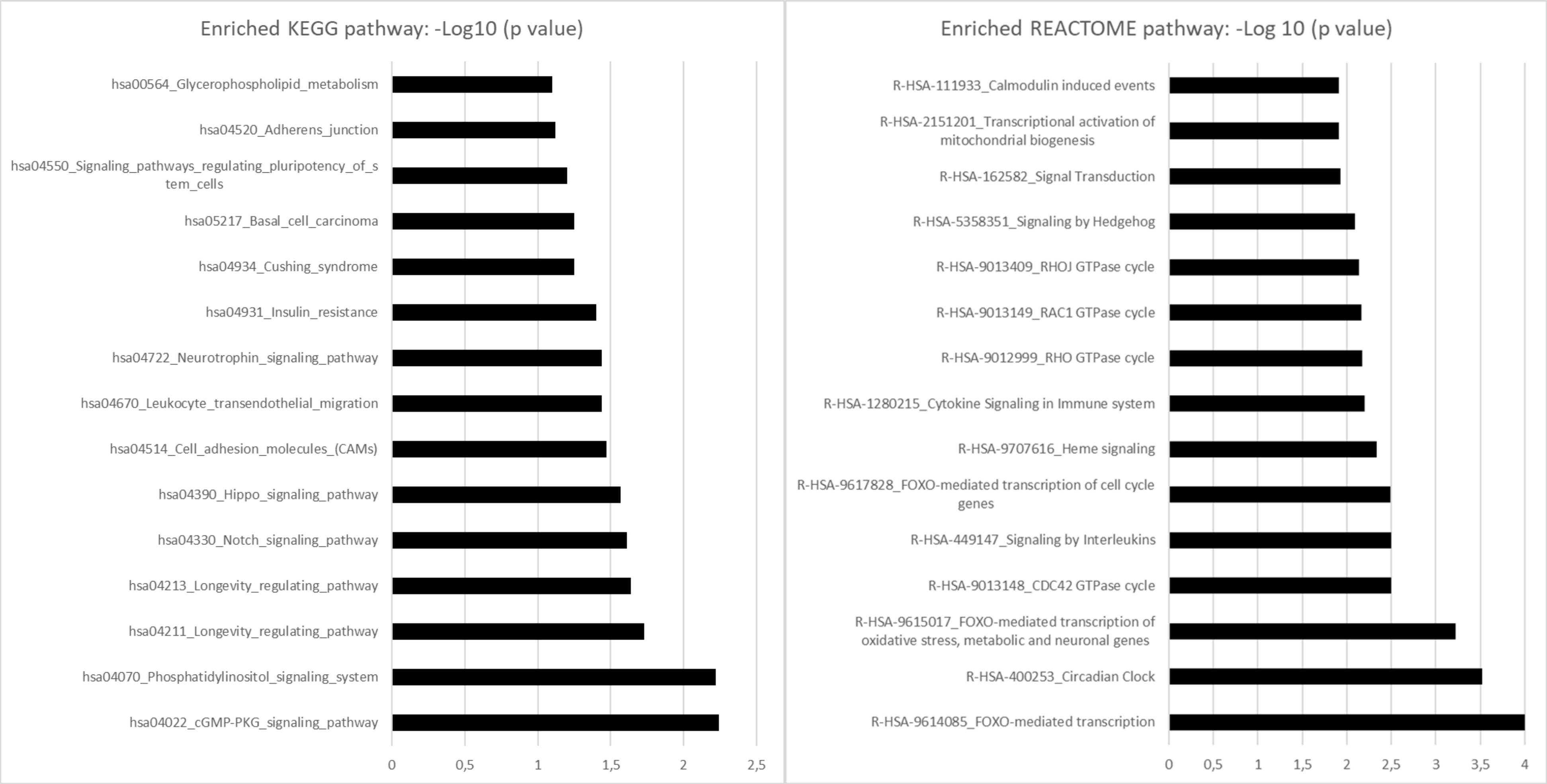

Supplemental Figure S1. The top 15 enriched KEGG and REACTOME pathways from predicted target genes of miR-21-5p and miR-126-3p searched by miRWalk.

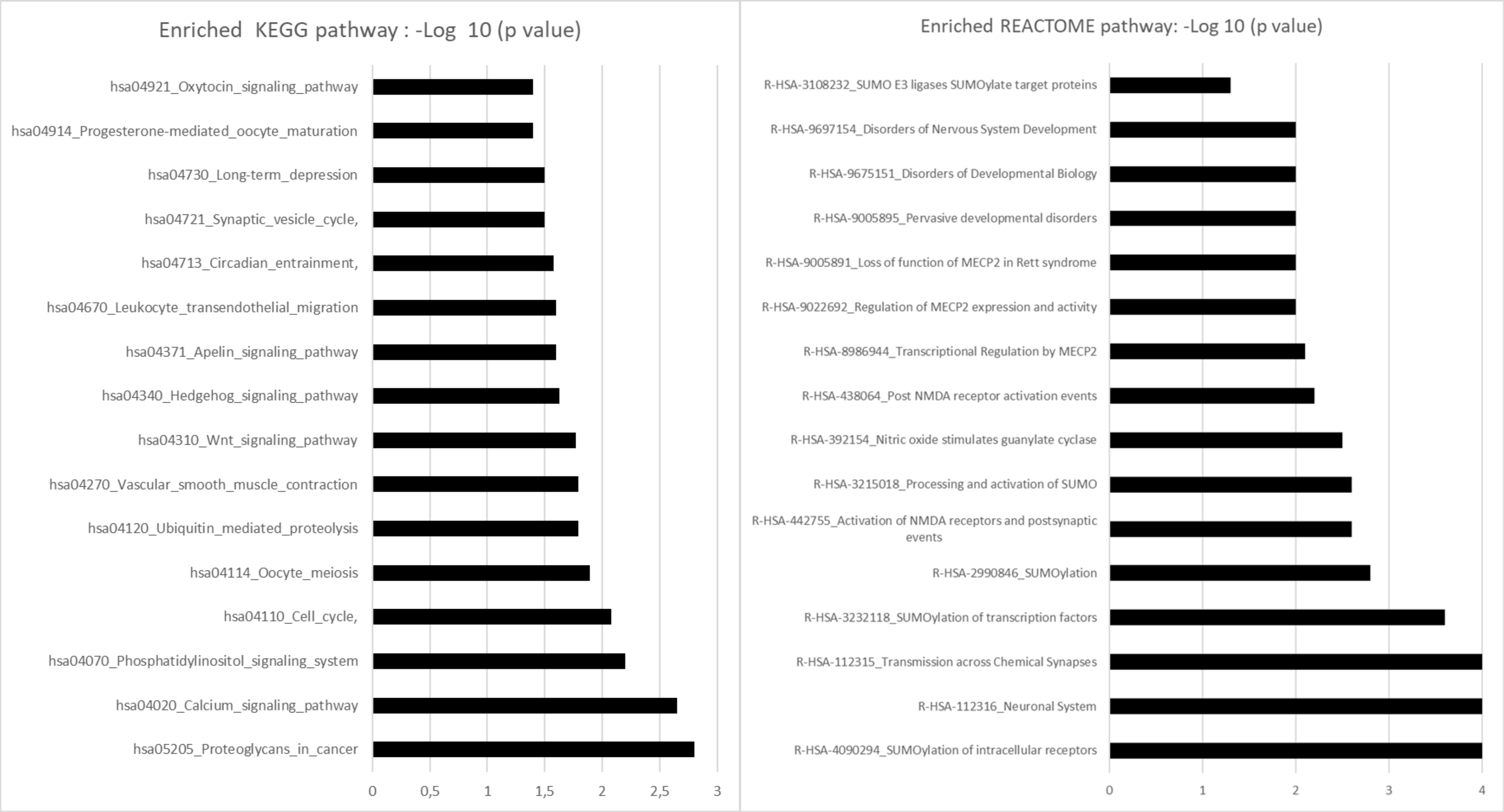

Supplemental Figure S2. The top 15 enriched KEGG and REACTOME pathways from predicted target genes of miR-146a-5p and miR-181a-5p searched by miRWalk.
